# Theoretical Study of High-Order Velocity Focusing Achieved with Single-Stage Reflectron Time-of-Flight Mass Spectrometry

**DOI:** 10.1021/jasms.5c00167

**Published:** 2025-11-26

**Authors:** Yi-Hong Cai, Yi-Sheng Wang

**Affiliations:** Genomics Research Center, 38017 Academia Sinica, Taipei 115, Taiwan

**Keywords:** coupled space and velocity focusing, reflectron time-of-flight mass spectrometry, single stage, delay extraction, flight-time topology

## Abstract

This study explores unexplained fundamental principles of reflectron time-of-flight (R-TOF) mass spectrometry (MS). Conventional calculations focusing on the ion trajectory in reflectors concluded that multistage reflectors are necessary to achieve second-order velocity focusing at ion detectors. This study demonstrates that in an instrument equipped with a matrix-assisted laser desorption/ionization (MALDI) ion source a single-stage reflector can achieve second-order velocity focusing when the optimal experimental parameters predicted using the coupled space and velocity focusing (CSVF) principle are used. The optimization model indicates that the delayed extraction technique is more effective in compensating for the initial ion velocity spread than reflectors. The calculation shows that for ions with *m*/*z* of 10,000, the predicted maximum mass resolving power (*R*_*m*_) can reach 750,000 using a single-stage R-TOF MS with an effective total length of about 2.4 m, or approximately 130,000 when accounting for the temporal response limit of ion detectors. The calculation model also reveals that in second-order focusing conditions, ions have two focusing points along the flight path, instead of just one at the detector as conventionally believed. The result indicates that the new model is critically important for the advancement of R-TOF MS.

## Graphical abstract

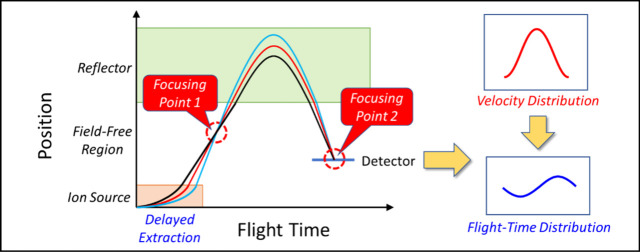

## Introduction

Advancing the analytical performance of mass spectrometers is important for applications demanding high-quality data. Particularly, the mass resolving power (*R*_*m*_) of time-of-flight (TOF) mass spectrometry (MS) is a critical feature for accurate molecular analysis. Currently, reflectron (R)-TOFMS remains one of the key methods in MS analysis because reflectors can reduce nonideal instrumental factors, so as to achieve high-order ion focusing by compensating the energy difference of ions and extending ions’ flight distances and times to increase *R*_*m*_.^1–3^ Nowadays, a typical R-TOFMS can offer a *R*_*m*_ between 10,000 and 100,000.^4,5^ The basic principle of R-TOFMS is also the foundation for the development of multiturn and multireflection TOF MS systems, which are advanced variants of R-TOFMS.^5,6^

The *R*_*m*_ in R-TOF MS is mainly affected by nonideal factors in the ionization process, such as those in electrospray ionization (ESI) or matrix-assisted laser desorption/ionization (MALDI) ion sources.^7,8^ The primary factors include (1) the initial spatial spread and (2) the initial velocity spread of ions.^9–12^ The initial velocity spread is particularly critical in the sense that ions with different initial velocities reach the detector at different times. The primary concept of reflectors to minimize their impact on *R*_*m*_ is allowing ions with higher initial velocity to spend a longer time in the reflector than those with lower initial velocity, thus achieving time focusing. Further instrumental optimization of reflectors to achieve high-order time focusing has been developed mainly by two significant advances, including the changes of the electric field from single-stage to (1) two-stage or (2) nonlinear (i.e., parabolic) configurations.^1,13–16^ Most of these advancements considered velocity focusing in TOFMS using a simplified formula and ignored the impact of spatial spreads.^1,9,10,12^ Formula simplifications are generally achieved by neglecting terms with minor influence, thereby making the equations more concise and facilitating the calculation with limited computational resources. However, under ultrahigh-resolution conditions, every term becomes critical. Based on the simplified models, only two-stage linear or parabolic reflectors can achieve second- and infinite-order focusing, respectively.^2,9,14^ However, the simplified models may hinder the understanding of true ion behavior and deliver inaccurate results.^14,16,17^

Calculations using the full flight-time formulation are desirable but challenging. The space-velocity correlation focusing^18^ and coupled space and velocity focusing (CSVF) methods^19,20^ are two examples that use the full flight-time calculation function for prediction. Both methods consider the relevance between velocity and spatial focusing conditions in linear TOFMS. We have described the comprehensive CSVF model and the calculation technique thoroughly in the past, which considers a group of ions to mimic real experimental conditions.^18,21^ The calculations employ the full flight-time expression and numerical analysis to comprehensively account for all variables and their full range of possible values. Based on the calculation, the *R*_*m*_ of linear-TOFMS can be enhanced significantly by optimizing instrumental and ion extraction parameters. The CSVF model can be applied to other TOF analyzers, as long as the flight time equation is precisely derived. Nonetheless, these methods have not been applied to optimize R-TOFMS owing to the instrument’s complex structure.

This study utilizes the CSVF model to analyze first- and second-order velocity focusing in a single-stage MALDI-R-TOF mass spectrometer. The model can accurately predict the *R*_*m*_ and reveals important unaware ion behavior that is critical for the instrument’s performance. In the case of second-order velocity focusing, the ion group has two spatial focusing points along its flight path rather than a single focal point at the detector. The calculation results show that the delayed extraction technique is more effective in compensating for the initial velocity spread of ions than the reflector. The results show that ions of *m*/*z* of 10,000 can reach the maximum *R*_*m*_ of 750,000 in the optimal condition, or approximately 130,000 when accounting for the temporal response limit of ion detectors. The flight-time distributions are carefully discussed, revealing critical interesting facts that we were not aware of in the past. The analysis of ions with an *m*/*z* of 1,000 is also included. These findings highlight that a comprehensive understanding and precise modeling of the flight-time expression of ions are prerequisites for unlocking the full performance of reflectron TOFMS.

## Experimental Section

The calculation considers an R-TOF mass spectrometer equipped with a MALDI ion source (Figure 1). The dimensions of the instrument are adapted from a conventional instrument that has a total length (*L*) of 1,200 mm. The MALDI ion source resembles the Wiley–McLaren design that consists of a sample, an extraction, and a grounded electrode.^12^ The respective lengths of the ion extraction (*s*_*0*_) and acceleration (*d*) regions are 8 and 10 mm. The lengths of the first field-free drift region (*D*_*1*_), reflector (*r*), and second field-free drift region (*D*_*2*_) are 882, 300, and 800 mm, respectively. The reflector has a single-stage electric field generated using an entrance/exit electrode kept at a ground potential and an end electrode biased with a high voltage. We assume no field distortion at the interface of every section, such as when their openings are covered by metal mesh. The calculations considered the *R*_*m*_ of ions of *m*/*z* 1,000 (in Supporting Information) and 10,000 in both continuous extraction (CE) and delayed extraction (DE) modes. In the DE mode, the sample electrode is biased at +25 kV, and the grounded electrode and the drift tube are held at ground potential. For delayed extraction, the voltage of the extraction electrode is kept at +25 kV during laser excitation and switched to a lower voltage, ranging from +24.8 to +20 kV, after an extraction delay (τ), corresponding to an extraction voltage (*V*_*s*_) of 200 to 5,000 V. The acceleration voltage (*V*_*d*_) during ion extraction is thus the voltage difference between the extraction and grounded electrodes. In the CE mode, the sample and extraction electrodes are biased constantly at the final voltages employed during ion extraction in the DE mode. In both CE and DE modes, the voltage in reflector (*V*_*r*_) is kept constantly at +26 kV.

**Figure 1:**
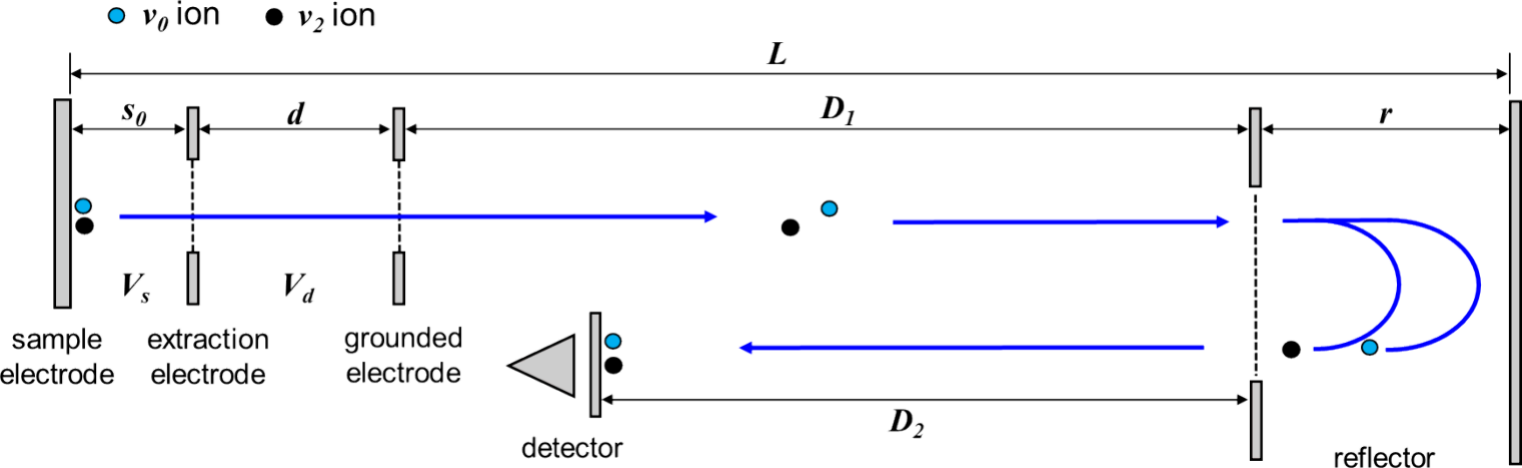
Schematic diagram of the MALDI R-TOF mass spectrometer discussed in this work. The blue and black circles are representative ions with initial velocities of *v*_*0*_ and *v*_*2*_, respectively.

### CSVF Model

The calculation model was developed based on the CSVF method described previously.^22,23^ The total flight time (*t*) of an ion (with mass *m* and charge *q*) includes the flight time the ion spends in the extraction (*t*_*s*_), the acceleration (*t*_*d*_), the first field-free drift (*t*_*D1*_), the reflector (*t*_*r*_), and the second field-free drift (*t*_*D2*_) regions. Assuming the ion’s initial velocity is *v*_i_, the general expression of *t* becomes

1 $$t\left(v_{i},\tau \right)=t_{s}+t_{d}+t_{D_{1}}+t_{r}+t_{D_{2}}=\frac{\sqrt{2m}}{qE_{s}}\left[{\left(U_{i}+U_{s}\right)}^{1/2}-{U_{i}}^{1/2}\right]+\frac{\sqrt{2m}}{qE_{d}}\left[{\left(U_{i}+U_{s}+U_{d}\right)}^{1/2}-{\left(U_{i}+U_{s}\right)}^{1/2}\right]+\frac{\sqrt{2m}D_{1}}{2{\left(U_{i}+U_{s}+U_{d}\right)}^{1/2}}+2\frac{\sqrt{2m}}{qE_{r}}\left[{\left(U_{i}+U_{s}+U_{d}\right)}^{1/2}\right]+\frac{\sqrt{2m}D_{2}}{2{\left(U_{i}+U_{s}+U_{d}\right)}^{1/2}}$$

Where *E*_*s*_ (=*V*_*s*_/*s*_*0*_), *E*_*d*_ (=*V*_*d*_/*d*), and *E*_*r*_ (=*V*_*r*_/*r*) are the electric fields in the extraction, acceleration, and reflector regions, respectively. The *U*_*s*_ (=*qs*_*i*_ *V*_*s*_, where *s*_*i*_ (=*s*_*0*_ – *τv*_*i*_) is the ion’s extraction length) and *U*_*d*_ (=*qdE*_*d*_) are respectively the potential energies in the extraction and acceleration regions, and *U*_*i*_ represents the ion’s initial kinetic energy (=1/2 *mv*_*i*_ ^2^). The calculation considers the ions within the full width at half-maximum (fwhm) of the Maxwell–Boltzmann velocity distribution under typical MALDI conditions. In the current study, the discussion is based on 2,5-dihydroxybenzic acid matrix ionized by a pulsed UV laser beam, producing a typical desorption temperature of 1700 K.^22,24,25^ Under these conditions, the ions with two representative *v*_*i*_s defining the fwhm are 167.42 (*v*_*2*_) and 878.23 (*v*_*0*_) m/s. The 878.23 m/s ion is assigned the reference ion, and only the integer values of the velocities will be mentioned hereafter. The initial ion velocity range may vary depending on specific experimental conditions or applications. The method for optimizing the instrument is primarily based on eq 2, which represents the flight-time spread (Δ*t*) between the two ions.^23^

2 $$t\left(v_{2},\tau \right)-t\left(v_{0},\tau \right)\approx 0$$

Solving eq 2 finds the τ(s) that allow the two ions to reach the detector’s surface simultaneously. That is, the optimal τs adjust the ions’ total kinetic energy to compensate for their initial kinetic energy difference. In the current study, the optimal τs making eq 2 approaches zero, or below 10^–13^ were obtained by numerical analysis. Such a condition is defined by satisfying eq 2 hereafter. If eq 2 does not hold with any τ, the τ that results in the minimum value of eq 2 is determined. The calculations produced large data sets for subsequent big data processing and detailed topological analysis.

### Flight-Time Distribution (Topology)

Once the optimal τ value is determined, the flight time can be calculated using eq 1. The maximum flight-time difference Δ*t*_*max*_ = (*t*_*max*_ – *t*_*min*_) and the central flight time *t*_*c*_ = (*t*_*max*_ + *t*_*min*_)/2 in the initial velocity range of 167–878 m/s will be used to calculate the *R*_*m*_ (=*t*_*c*_/(2Δ*t*_*max*_)). The topological analysis or flight-time distribution in the current work considers all ions within the fwhm of the Maxwell–Boltzmann distribution. In the calculated flight-time distributions in this work, every data point is obtained by using the best τ (determined by eq 2). The analysis focuses on the change in *R*_*m*_ for various types of flight-time distributions.^19^ All calculations are performed using Mathematica 10 (Wolfram Research, Champaign, IL, USA), and the results showed negligible differences (<0.00009%) with those obtained by SIMION 8.0.

## Results and Discussion

### Optimal Extraction Voltage and Delay

The calculation systematically analyzed the ion behaviors in every part of the instrument. To evaluate the importance of the ion source and reflector parameters, the simulation compares the result obtained using CE and DE modes. The discussion herein focuses on the results for ions of *m*/*z* 10,000 because they show similar trends as those of *m*/*z* 1,000 and the variations are more pronounced. The corresponding results for ions of *m*/*z* 1,000 are summarized in Supporting Information S1–S7.

For ions of *m*/*z* 10,000, the calculation results show that the extraction voltage has a substantial effect on the *R*_*m*_. When scanning the *V*_*s*_ from 200 to 5,000 V, the *R*_*m*_ in DE mode exhibited a main peak of above 750,000 at 3,635 V and a minor peak of roughly 175,000 at 3,182 V, as shown in Figure 2a. Detailed analysis indicates that the ions can achieve first-order time focusing effect with an appropriate τ (that satisfies eq 2) when above 2,794 V. At this critical voltage, the obtained Δ*t* is about 45.91 ns, corresponding to an *R*_*m*_ of roughly 1,437. On the other hand, the ions achieve second-order focusing within 3,036–3,235 V and 3,541–3,693 V, which correspond to the minor and main peak regions, respectively. When the *V*_*s*_ exceeds 3,693 V, the ion focusing effect only achieves first-order velocity focusing, and the achievable *R*_*m*_ range is approximately 42,000 to 520,000, as indicated in Figure 2a. On the other hand, the analysis of τ utilized in Figure 2a shows two critical voltages at 2,279 and 2,867 V, as shown in Figure 2b. Below 2,279 V, τ needs to be 0 in order to achieve the lowest Δ*t*, indicating that the *V*_*s*_ is too low to make any improvement in DE mode. Under this condition, applying a nonzero τ increases Δ*t*, suggesting that the ions must gain kinetic energy from the full electric (or potential) energy in this region to achieve the minimum Δ*t*. When *V*_*s*_ is between 2,279 and 2,867 V, increasing τ gradually reduces Δ*t*. When *V*_*s*_ = 2,867 V, the best extraction length for reference and *v*_*2*_ ion is respectively 2.1 and 6.9 mm away from the extraction electrode. At this point, τ is at its peak value of 6.7 μs. Beyond 2,867 V, the best τ starts to decrease with increasing *V*_*s*_.

**Figure 2:**
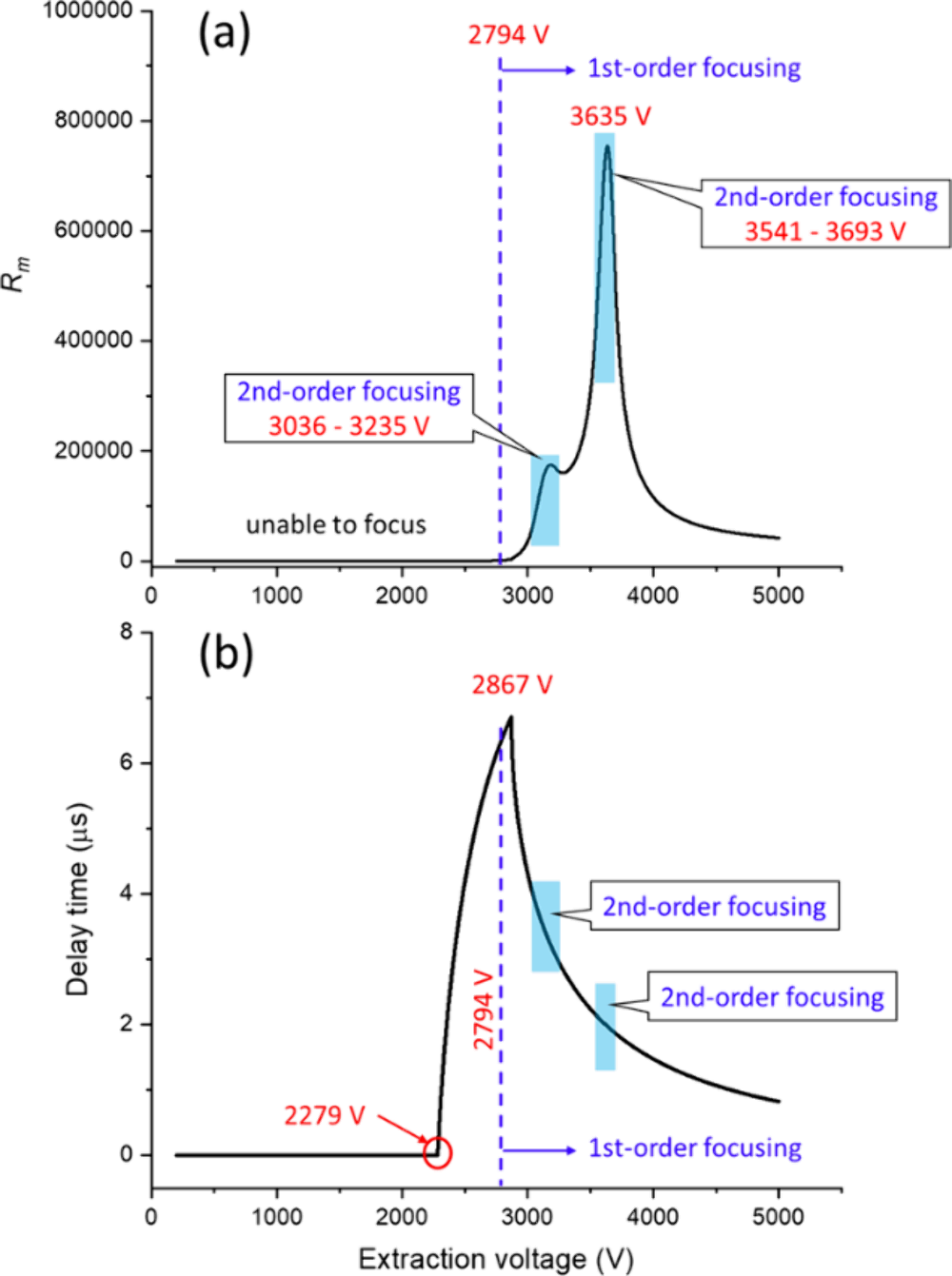
(a) *R*_*m*_ of ions of *m*/*z* 10,000 calculated with various extraction voltages. (b) Optimal delay time of the same ions calculated with various extraction voltages.

The calculation result indicates that the difference in the electric energy gain by the ions is effective for counterbalancing the ions’ initial energy spread. During this process, τ plays a key role because it determines the extraction length as well as the electric energy the ions gain. The compensation of initial energy difference by some variants of post acceleration devices are also realized in imaging mass spectrometry with R-TOF mass spectrometer to improve mass resolution while preserving spatial resolution.^26–29^

### Initial and Terminal Velocities of Ions

The initial and terminal velocities of ions with the same *m*/*z* as they leave the ion source are major factors determining the effective time focusing. The terminal velocity of ions greatly influences their flight time in various regions, particularly in the reflectron. To thoroughly understand the ion behavior, the study compares the results in the CE mode and the DE mode under optimal conditions.

#### CE Mode

Under this mode, all ions gain the same electric energy because *V*_*s*_ are activated prior to ion generation at the sample surface. When ions leave the ion source and enter the first field-free region, the terminal velocity of ions is positively correlated with the initial velocity. That is, ions with a lower initial velocity have a lower terminal velocity, and vice versa. Figure 3 shows the result when *V*_*s*_ = 3,635 V, which is the best *V*_*s*_ in DE mode. Under this condition, the *v*_*2*_ (167 m/s) and reference (878 m/s) ions respectively show the terminal velocities of 21,965 and 21,982 m/s. It is noteworthy that the relative velocity between *v*_*2*_ and the reference ions reduced significantly from −711 (initial) to −17 m/s (terminal) after gaining electric energy in the ion source. It is impossible, however, for ions with a lower initial velocity to have a higher terminal velocity under this mode.

**Figure 3:**
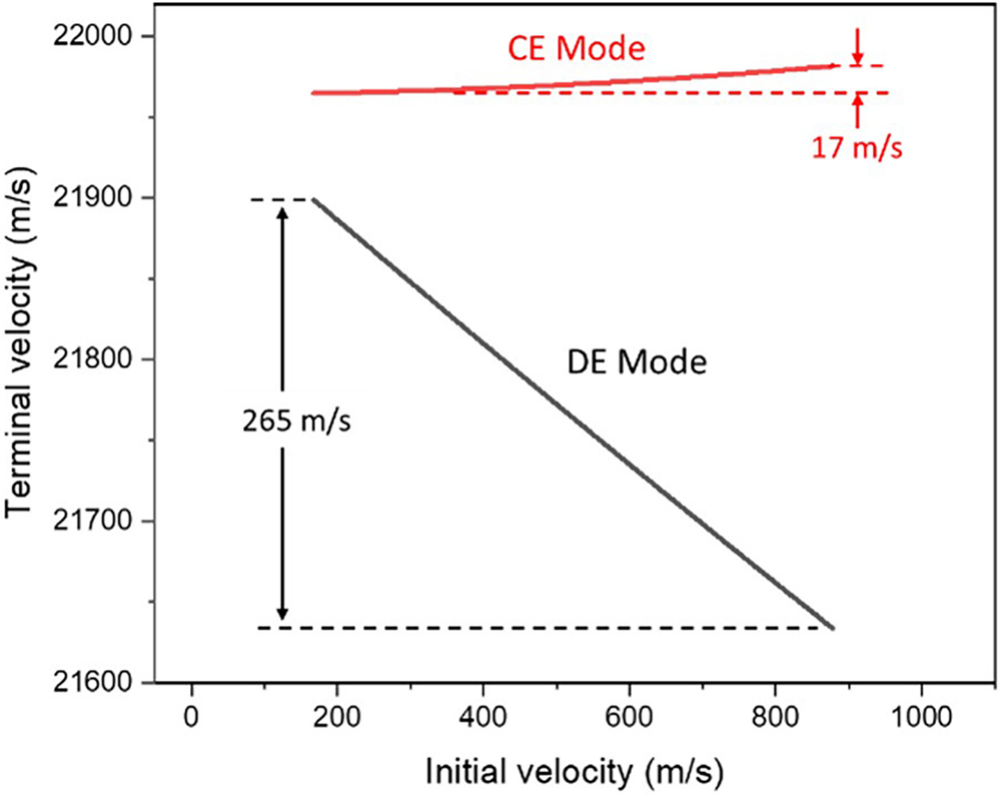
Relationship between the initial and terminal velocities of *m*/*z* 10,000 ions.

#### DE Mode

Under the DE mode, ions with a lower initial velocity depart from the surface with a lower speed. Therefore, the electric field imparts more kinetic energy to these ions in the extraction region when the *V*_*s*_ activated after some delays. With an appropriate τ, an initially slower ion can achieve a higher terminal velocity. Figure 3 depicts the optimal condition under DE mode where *V*_*s*_ = 3,635 V and τ = 1,972 ns, in which the *v*_*2*_ (167 m/s) and reference (878 m/s) ions respectively show terminal velocities of 21,899 and 21,634 m/s. That is, the relative velocity changes from −711 (initial) to +265 m/s (terminal). In fact, the DE method not only reverses the correlation between initial and terminal velocities but also allows fine adjustment of the terminal velocity difference to optimize the ion focusing effect. Therefore, it provides an opportunity to reduce or eliminate the Δ*t* of the two ions.

### Flight-Time Distribution

The flight-time distributions in CE and DE modes reveal, respectively, how important the reflectron and delayed extraction techniques are to improve *R*_*m*_. The calculation discusses the distributions systematically in every region to show their changes across the entire instrument. Notably, the current study presents the results of the best *R*_*m*_ in both modes; therefore, the results change as parameters change. Conventional models based on simplified assumptions and equations are unable to provide accurate results to mimic real ion behaviors.^1,9,12^

#### Extraction Region (*t*_*s*_)

Ions start the journey from the extraction region, and this is the only region allowing users to adjust the total energy by changing the τ. The flight-time distribution within this region in the CE mode is illustrated in Figure 4. The result shows that the *t*_*s*_ and initial velocity have an inverse linear correlation. The respective *t*_*s*_ values for *v*_*2*_ and reference are 1.873 and 1.721 μs, corresponding to the *v*_*2*_ ion lagging behind the reference by approximately 152 ns. Under DE mode, the *t*_*s*_ values show a similar trend except that the *v*_*2*_ ion falls further behind to approximately 330 ns. The stronger correlation in DE mode is due to the fact that the ions are already away from the sample surface with different extents when the extraction voltage activated after τ. Because the extraction lengths (=*s*_*0*_ – *v*_i_*τ) for ions with higher initial velocity are shorter (closer to the extraction electrode), they can reach the end of the extraction region earlier. Although the initially slower ions can gain higher electric energy (because they are closer to the sample electrode), its contribution on reducing *t*_*s*_ is insignificant since the extraction region is too short to reduce their gap with the reference ion.

**Figure 4:**
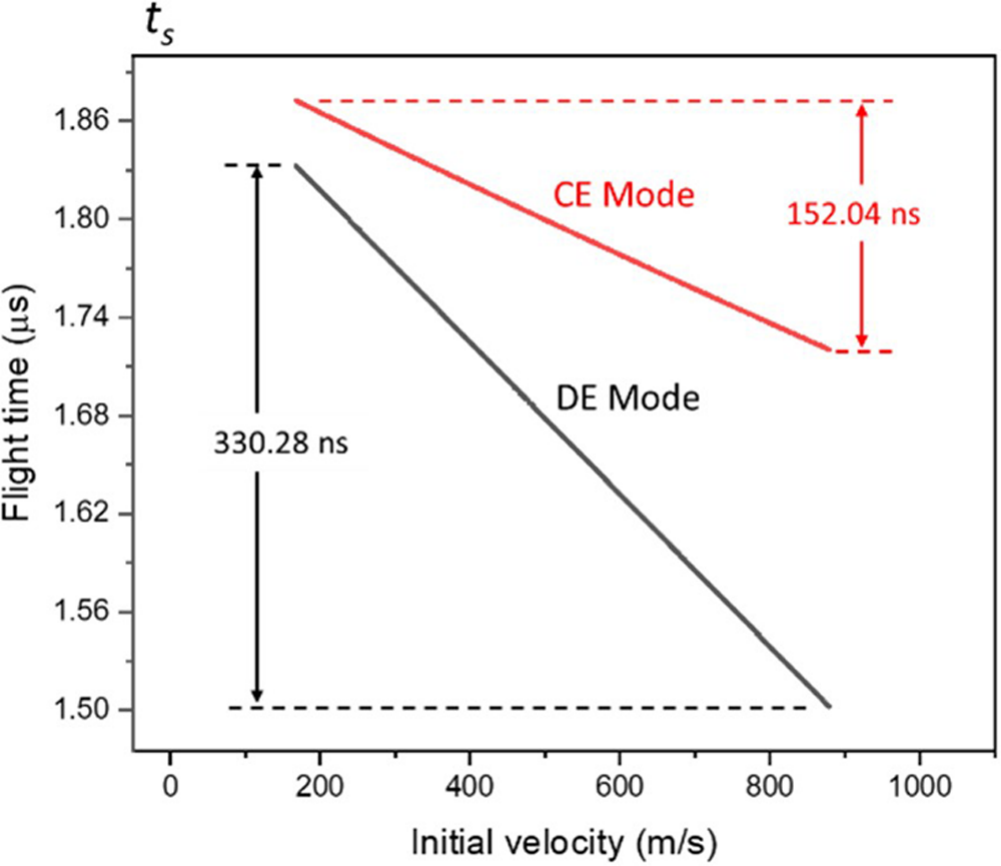
Flight-time distribution of ions in the extraction region.

#### Acceleration Region (*t*_*d*_)

Either in CE or DE mode, all ions gain the same electric energy across this region regardless of their initial velocity. Although the acceleration region is only 2 mm longer than the extraction region, the ions’ *t*_*d*_ is 1/3 that of their *t*_*s*_ because ions are already accelerated in the extraction region, as shown in Figure 5. In the CE mode, the *t*_*d*_ shows the similar inverse correlation with initial velocity as that in Figure 4, but the maximum *t*_*d*_ difference with respect to the reference ion is only about +1.33 ns. In DE mode, in contrast, *t*_*d*_ shows a direct correlation with initial velocity, indicating that initially slower ions fly across this region faster than initially faster ions, which is opposite to the curve in Figure 4. The inversion is due to the fact that an initially slower ion already developed a higher velocity than initially faster ions when entering this region, which subsequently results in a relatively smaller *t*_*d*_. The maximum *t*_*d*_ difference with respect to the reference ion under this condition becomes approximately −22.89 ns. For an ion with a specific initial velocity, the smaller *t*_*d*_ value in CE mode in comparison to DE mode is due to the fact that ions are imparted with the complete electric energy in the extraction region in CE mode but less energy in DE mode owing to the change in the extraction length.

**Figure 5:**
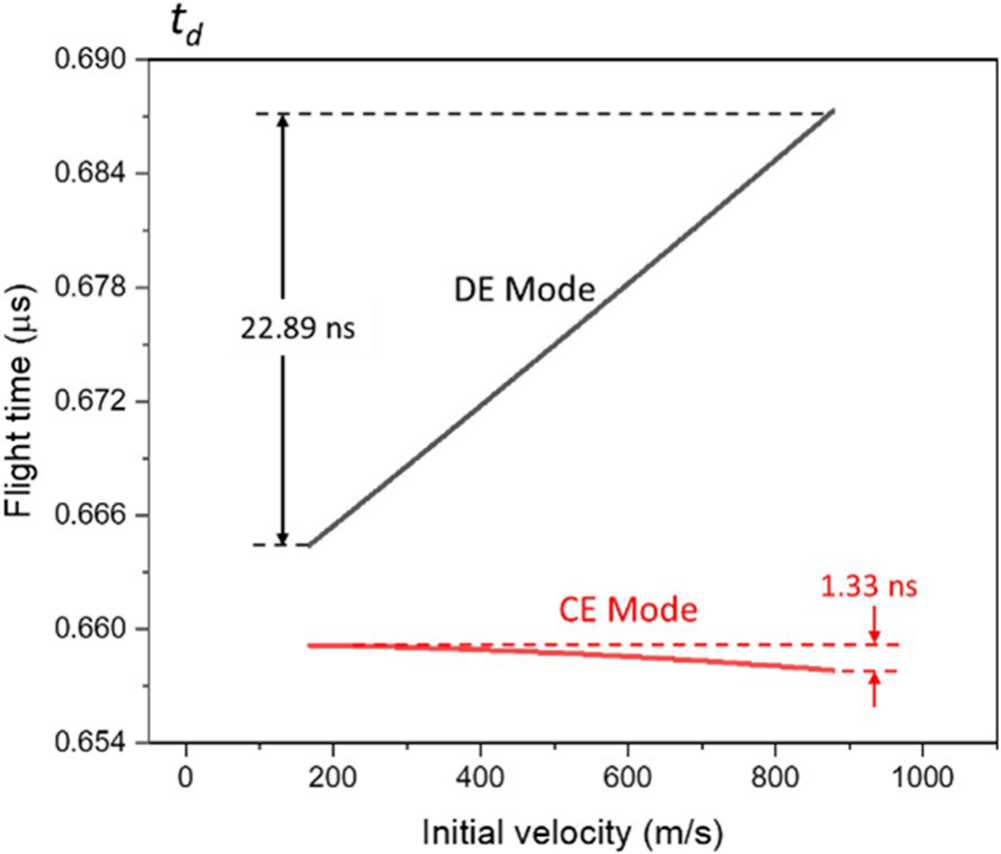
Flight-time distribution of ions in the acceleration region.

It is noteworthy that ions reach their terminal velocity (Figure 3) when they pass the grounded electrode. The overall impact of the electric potential up to this point can also be evaluated using the arrival time at the grounded electrode or *t*_*s*_ + *t*_*d*_. With respect to the reference ion, the maximum flight-time spread in the extraction and acceleration regions, or Δ*t­(s+d)*, is approximately +153.37 ns in CE mode and +307.39 ns in DE mode. That is, the maximum flight-time difference up to this point in DE mode still shows a much worse time-focusing effect than CE mode.

#### First Field-Free Region (*t*_*D1*_)

When ions leave the ion source, their terminal velocities determine the flight times in the rest of the regions. Since ions will travel at a constant velocity in field-free regions, their flight times can be calculated conveniently and precisely. Figure 6 illustrates the flight-time distributions when ions reach the entrance of the reflectron, corresponding to *t*_*s*_ + *t*_*d*_ + *t*_*D1*_. Under CE mode, the data curve shows a trend similar to those in Figures 4 and 5; the distribution is inversely correlated to initial velocity, and the maximum Δ*t*(*s* + *d* + *D1*) increases to +184.26 ns. In contrast, the distribution in DE mode shows a direct correlation with initial velocity with the maximum Δ*t*(*s* + *d* + *D1*) value of −186.53 ns. That is, the arrival time of ions with the slowest initial velocity is 186.53 ns shorter than the initially fastest (reference) ions.

**Figure 6:**
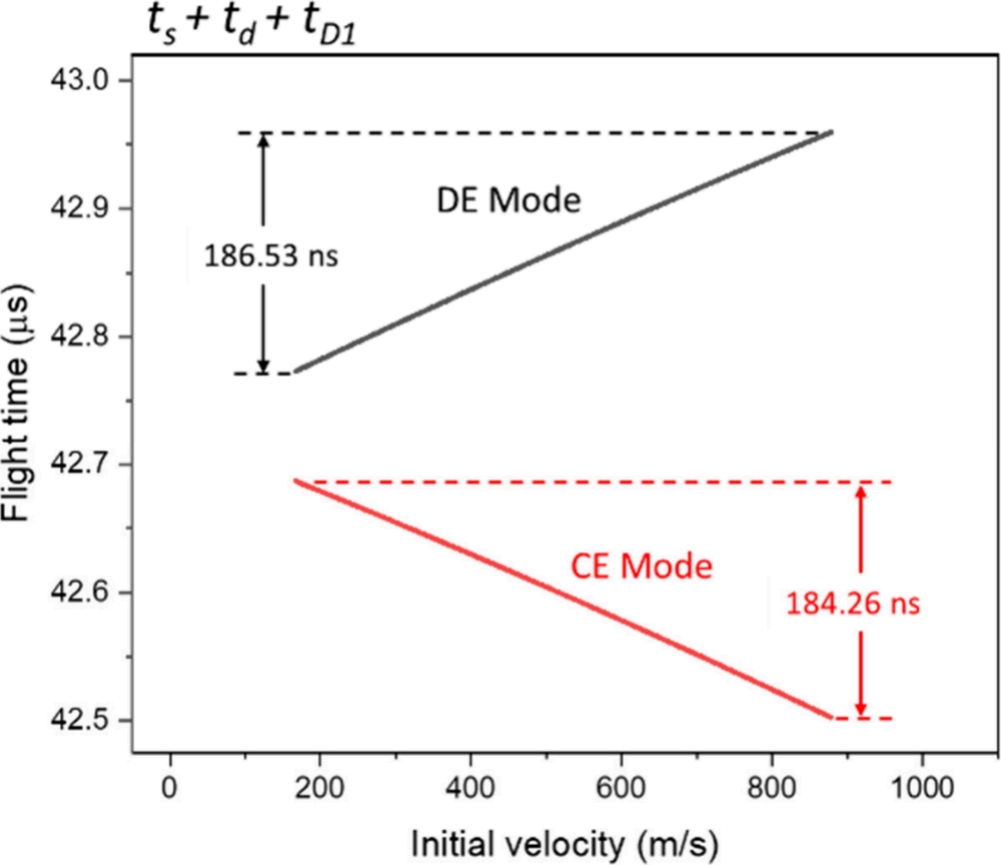
Flight-time distribution of ions reaching the end of the first field-free region.

The change from a positive Δ*t*(*s* + *d*) value to a negative Δ*t*(*s* + *d* + *D1*) value indicates that the *v*_*2*_ ion passed by the reference ion in this region. That means the experimental condition generates a space focusing point in this region. The presence of a focusing point also inverse the slop of the overall flight-time distributions from a negative to a positive trend.

#### Reflector Region (*t*_*r*_)

The main purpose of reflectors is to reduce the flight-time spread between ions with distinct initial velocities. The focusing effect is achieved by making high-speed ions spend longer times in the reflector than low-speed ions. It needs to emphasize that the *t*_*r*_ is determined by ions’ terminal velocity, instead of initial velocity. The resultant *t*_*r*_ is in the range of 50–53 μs in both CE and DE modes, as shown in Figure 7. The long *t*_*r*_ values are due to the ions’ deceleration and reacceleration process induced by the retarding field in the reflector. It needs to be stressed that conventional approaches to compensate for the overall difference in *t*_*s*_, *t*_*d*_, *t*_*D1*_ and *t*_*D2*_ mainly rely on Δ*t*_*r*_, but there are few variable parameters in typical reflectors.

**Figure 7:**
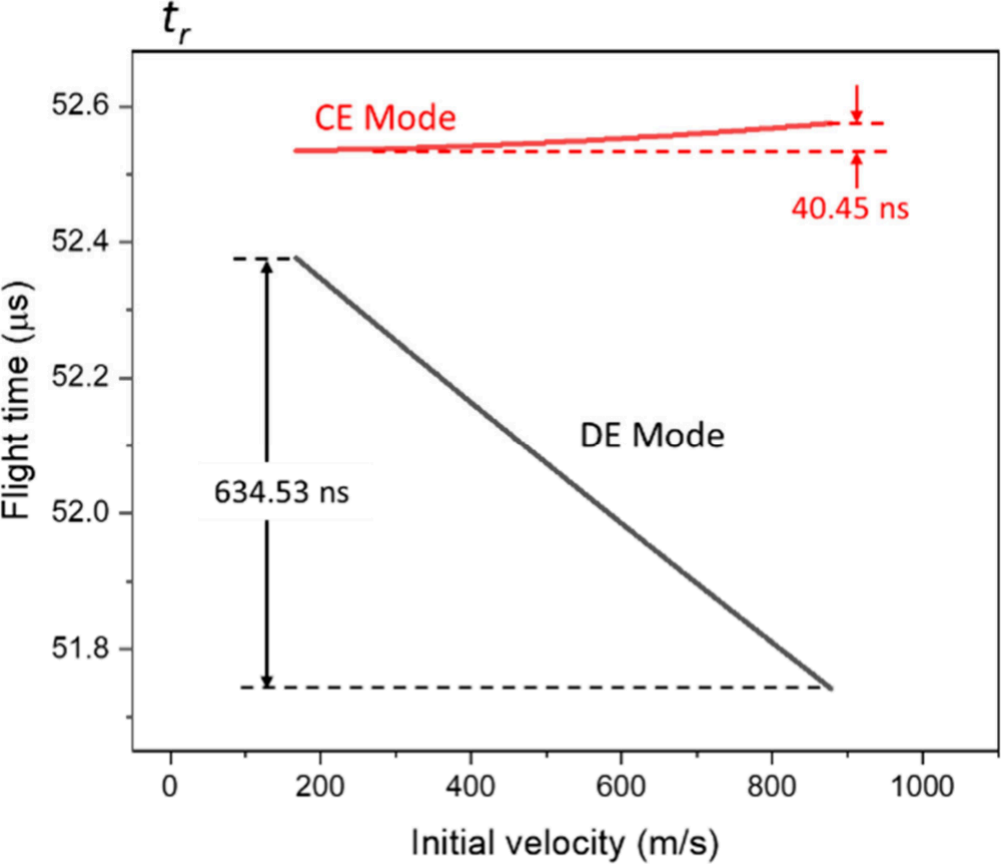
Flight-time distribution of ions in the reflector.

The maximum Δ*t*_*r*_s in the CE and DE modes are considerably distinct. In CE mode, the maximum Δ*t*_*r*_ value is −40.5 ns. This is the first and the only region that shows direct correlation between the flight-time distribution and the initial velocity in CE mode. However, such a Δ*t*_*r*_ is still much less than the maximum Δ*t*(*s* + *d* + *D1*) of +184.26 ns, suggesting that the time focusing effect is limited. In contrast, the maximum Δ*t*_*r*_ in the DE mode is 634.5 ns, which is much greater than the maximum Δ*t*(*s* + *d* + *D1*) of −186.53 ns in the DE mode. The significant *t*_*r*_ difference in DE mode is due to the 265 m/s terminal velocity difference between the initially slowest (*v*_*2*_) and fastest (reference) ions shown in Figure 3, when they leave the ion source.

The result shows that it is difficult to compensate for the flight-time differences caused by the initial velocity differences solely by using a reflector. A simple evidence is the insignificant *t*_*r*_ difference in CE mode due to merely 17 m/s terminal velocity difference between the two ions, as shown in Figure 3. The resultant Δ*t*_*r*_ in CE mode is even too less to compensate Δ*t*_*s*_ (the red line in Figure 4). In contrast, the results in DE mode suggest that it is possible to generate a large Δ*t*_*r*_ by combining delayed extraction and reflector to compensate the *t*_*s*_, *t*_*d*_, *t*_*D1*_ and *t*_*D2*_ differences.

It is worth mentioning that the function of the reflector becomes useful only if it is used in DE mode. The reason is that the ions’ terminal velocity and hence their *t*_*r*_ difference can only be optimized by adjusting τ. That is, the DE method provides the opportunity to reduce the overall flight-time difference of *t*_*s*_ + *t*_*d*_ + *t*_*D1*_, or even *t*_*s*_ + *t*_*d*_ + *t*_*D1*_ + *t*_*D2*_ to find the optimal focusing condition. The arrival time (*t*_*s*_ + *t*_*d*_ + *t*_*D1*_ + *t*_*r*_) distribution of ions as they reach the exit of the reflector is shown in Figure 8. In CE mode, the maximum arrival time difference is +143.81 ns (Figure 8), which is slightly shorter than that at the entrance of the reflector (+184.264 ns, Figure 6). In contrast, under DE mode, the maximum arrival time difference becomes approximately +448 ns (Figure 8), which inverses the flight-time correlation at the entrance of reflector (Figure 6). That is, the maximum Δ*t*_*r*_ is greater than the maximum Δ­(*t*_*s*_ + *t*_*d*_ + *t*_*D1*_). The remaining 448 ns difference is used to compensate for the *t*_*D2*_ difference, as will be discussed in the next section.

**Figure 8:**
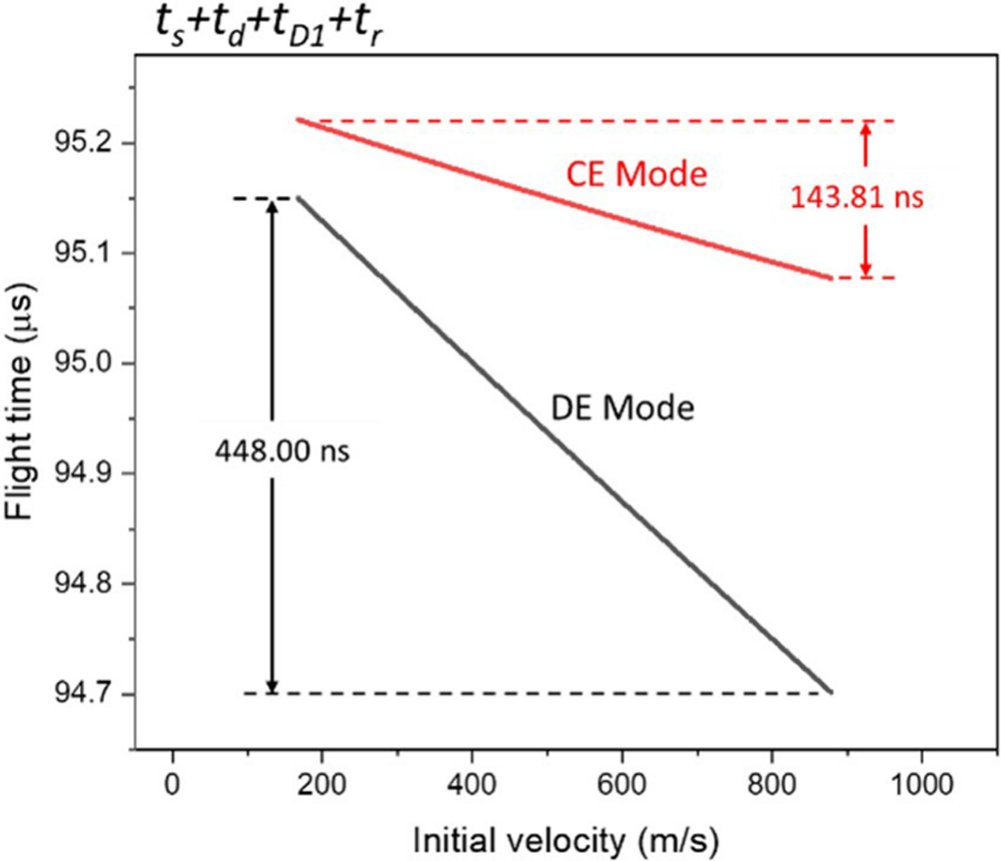
Flight-time distribution of ions reaching the exit of the reflector.

#### Second Field-Free Region

When the reflector is left, the ions enter the second field-free region and retain their terminal velocity (red line of Figure 3) again. The terminal velocities determine the flight time (*t*_*D2*_) in this region. In CE mode, the flight-time distribution is similar to that of *t*_*D1*_, in which the initially slower ions have a longer *t*_*D2*_, as shown in Figure 9. Precisely, the *v*_*2*_ ion has a *t*_*D2*_ of 36.42 μs, and the reference ion has a *t*_*D2*_ of 36.39 μs. The maximal Δ*t*_*D2*_ is about +28.02 ns.

**Figure 9:**
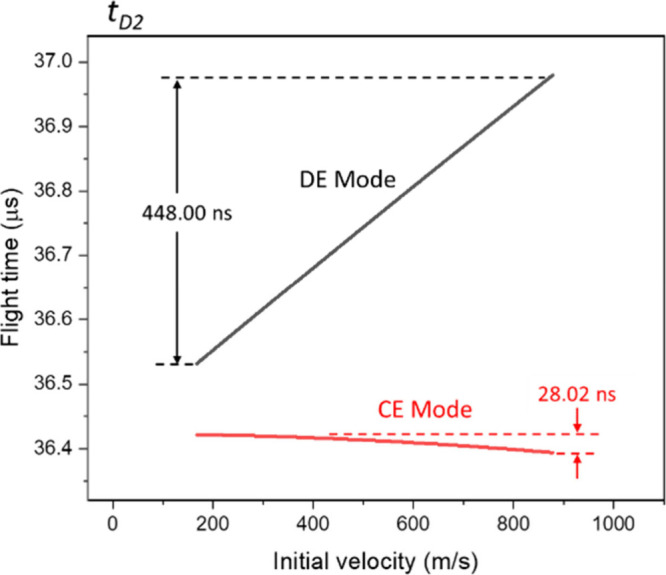
Flight-time distribution of ions in the second field-free region.

Under DE mode, due to the larger terminal velocity difference (Figure 3), the maximum *t*_*D2*_ difference reaches −448 ns. In this condition, the ion with slower initial velocity has shorter *t*_*D2*_. Notably, an important result in Figure 9 is that the maximum *t*_*D2*_ difference is the same as the arrival time difference at the exit of the reflector, except the correlation with ions’ initial velocity is inversed, as can be compared to the result in Figure 8. This facilitates the complete compensation of the flight-time difference of slow and fast ions at the detector surface.

One can now summarize the total flight time of the ions by calculating *t*_*s*_ + *t*_*d*_ + *t*_*D1*_ + *t*_*r*_ + *t*_*D2*_. Figure 10a displays the result obtained using the CE and DE modes. In the CE mode, the maximum arrival time difference increases from +143.81 ns in Figure 8 to +171.84 ns in Figure 10a, resulting in an *R*_*m*_ of about 383. In the DE mode, the flight-time distribution becomes a sinusoidal-like curve with two turning points (Figure 10b). This condition allows the initially 167 and 878 m/s ions to reach the detector at the same time. The overall Δ*t*_*max*_ is 0.09 ns, corresponding to an *R*_*m*_ of 754,891. The result indicates that the arrival time difference at the exit of the reflector (+448 ns, Figure 8) is eliminated almost entirely by the difference in *t*_*D2*_ (−448 ns, Figure 9), achieving a nice ion focusing effect at the detector surface. This condition satisfies CSVF that reaches second-order velocity focusing (with two turning points^19^). Notably, while the results offer new insights into the true ion behavior in reflectron TOFMS, the predicted maximum *R*_*m*_ is based on ideal conditions and remains unattainable at present due to several limiting factors. The most significant limitation is the detector’s temporal response limit (∼0.5 ns),^21,23^ which restricts the achievable *R*_*m*_ to approximately 130,000. Since experimental evidence has shown peak widths approaching the detector’s resolution limit, the impact of other factors on spectral quality, such as sample morphology,^20^ laser pulse width,^30,31^ or imperfect electric fields,^23^ remains unconfirmed and is currently considered to be less significant. However, when analyzing nonconventional samples or using harsh ionization conditions, the mass resolving power can still be substantially reduced, even under second-order velocity-focusing conditions. In addition, the optimal extraction conditions are *m*/*z*-dependent and should be reoptimized according to the target mass for the best performance. For instance, the *R*_*m*_ decreases respectively by about 38% or 45% when the *m*/*z* shifts by 100 around *m*/*z* 1000 and 10000, when the response limit of the detector is taken into account.

**Figure 10:**
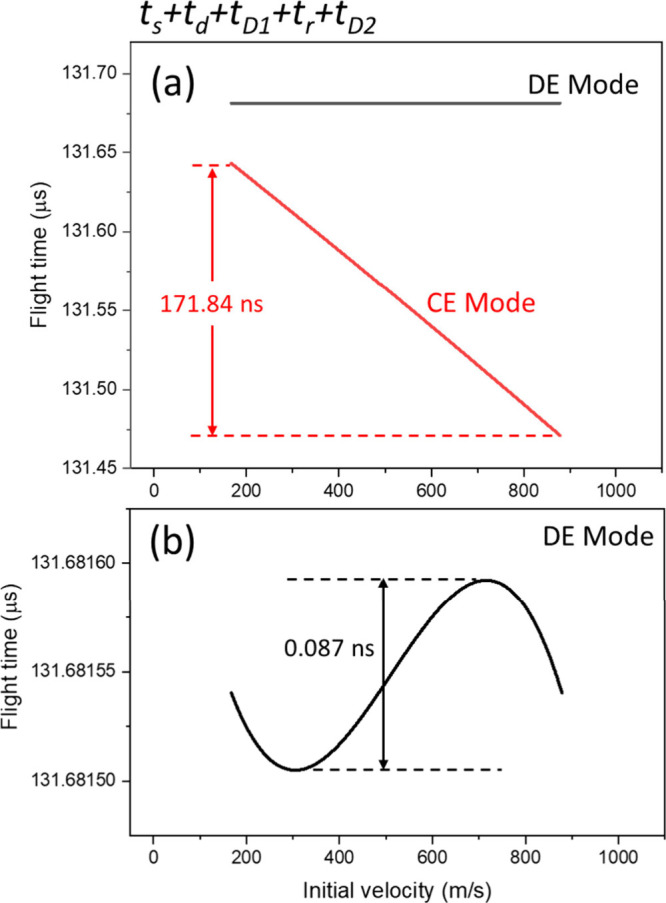
Flight-time distribution of ions when arriving at the detector: (a) regular range; (b) zoom-in to the black line in (a).

The results show that under optimized DE conditions, second-order velocity focusing for ions is still achievable with a single-stage reflector. Additionally, these results also indicate that the delayed extraction technique is more effective in compensating for the initial ion velocity spread than the reflectron technique. In the CE mode, the sequence of terminal velocity of ions is determined solely by their initial velocity. In this case, the terminal velocity differences between the representative ions are not significant (approximately 17 m/s). As a result, the reflector cannot effectively reduce the flight-time difference, unless the length of the reflector can be extended to a least 1.5 m.

In contrast to the CE mode, the difference in terminal velocities (265 m/s) in the DE mode can be adjusted by τ for the best flight-time focusing effect. That is, the performance of the reflector to adjust the flight-time difference within the reflector is mainly determined by τ. Therefore, the key to achieve ion focusing in R-TOF mass spectrometer should be the ion extraction parameters in the ion source.

#### Results of *m*/*z* 1000

The results for ions with *m*/*z* 1,000 are summarized in Supporting Information S1–S7. Under the same instrument configuration, the optimal extraction voltage for *m*/*z* 1,000 is approximately 3,800 V, obtaining a theoretical *R*_*m*_ of around 2.87 × 10^7^. When taking the detector’s response limit (∼0.5 ns) into consideration, the actual *R*_*m*_ limit reduce to approximately 40,000.

### Discussion

According to previous studies,^9,18,19^ the maximum *R*_*m*_ is determined by the order of velocity focusing. This order is defined by the power of the highest zero term in the Taylor expansion of the ion’s flight time expression. That is, to achieve the desired order of focusing, the corresponding term in the differential equation must be zero or approaches zero. For example, a second-order velocity focusing can be expressed as d^2^ *t*/d*v*_*i*_^2^ = 0. However, based on the CVSF model in the current study, second-order focusing conditions can be found without considering d^2^ *t*/d*v*_*i*_^2^ = 0. The results show that the flight-time distribution of ions with different initial velocities has a turning point in the case of first-order focusing, while it has two turning points in second-order focusing.

The results reveal two mysteries in R-TOF MS. Conventional R-TOF MS contain two consensuses: (a) the ions with faster initial velocity will reach deeper in the reflector and hence spend a longer time in the reflector region, and (b) the detector surface is the only ion-focusing point in the instrument. Our CVSF calculations indicate that, when achieving time focusing (satisfying eq 2), the initially faster ion indeed spends shorter time in the reflector. To clearly show the evolution of the flight-time spread, it is useful to compare the flight times of representative ions in every region. Figures S8–S12 illustrate the behavior of three representative ions with respect to their position and flight time in the CE and DE modes. The three ions are those with initial velocities of 167 (*v*_*2*_), 522, and 878 m/s (reference), in which 522 m/s is the peak value in the Maxwell–Boltzmann distribution. Since the differences are difficult to show macroscopically, Figure 11a,b summarizes the relationship qualitatively by emphasizing their differences. The results show that, in CE mode, the sequence of ions’ flight time remains unchanged except in the reflector region. That is, the flight times of the reference ion are always the shortest except in the reflector, but the reflector is unable to yield a long enough flight time to compensate for the overall difference. In DE mode, by contrast, initially slower ions spend longer time in the ion source but acquire higher terminal velocities, enabling them to overtake initially faster ions in the first field-free region and form the first focusing point. In the reflector, however, initially slower ions again lag behind faster ions at the exit. Owing to their higher terminal velocities, initially slower ions eventually catch up with initially faster ions at the detector surface, producing the second focusing point. That means that, under optimal DE conditions, the flight-time sequence reverses twice, ensuring that ions of different initial velocities arrive at the detector with minimum temporal spread. Therefore, two focusing points are generated in the second-order focusing condition rather than just one.

**Figure 11:**
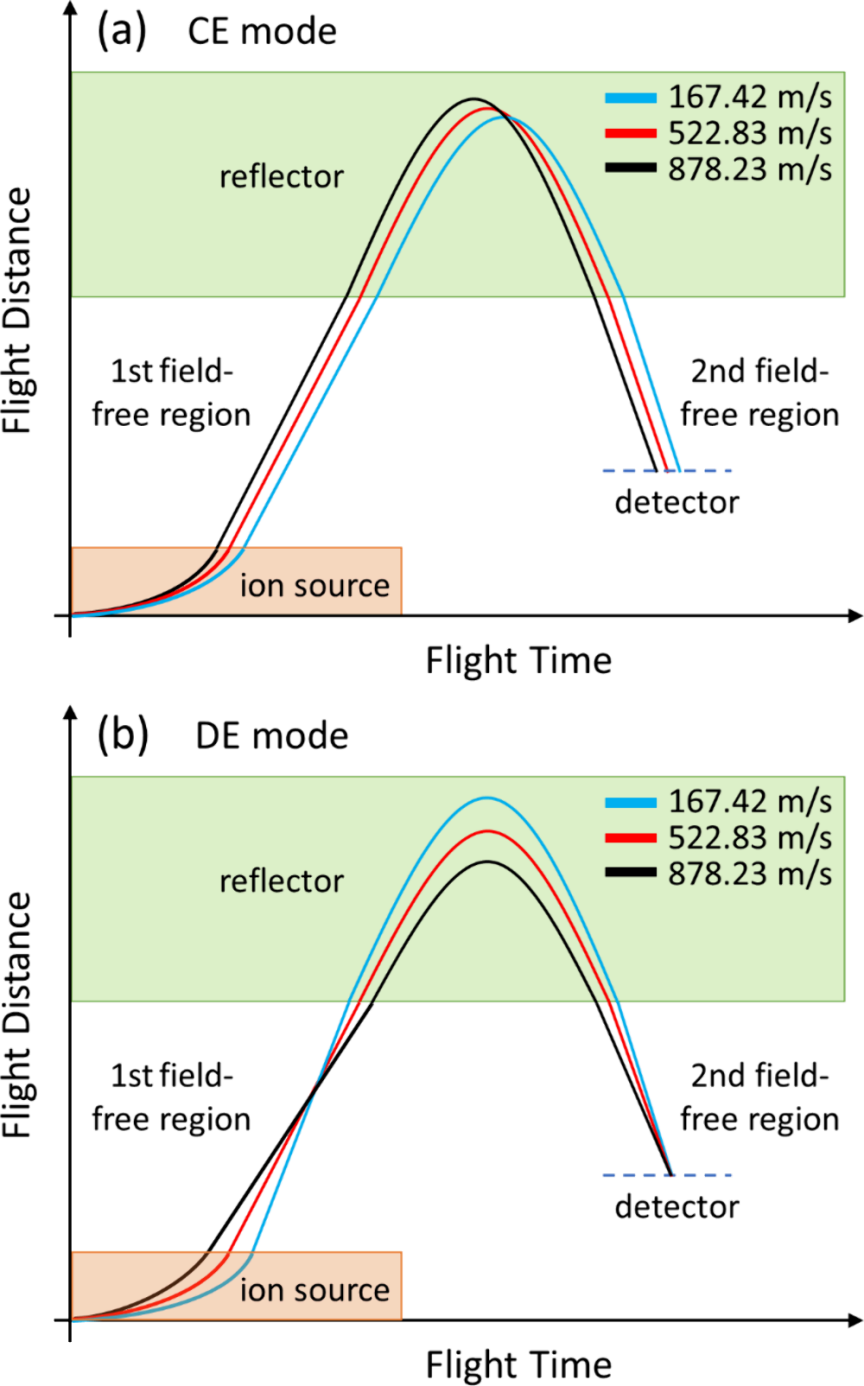
Correlation of flight distance and time across the entire instrument of representative ions with different initial velocities: (a) the result in CE mode; (b) the result in DE mode.

The findings of the current study indicate that achieving high-resolution results does not necessarily depend on sophisticated instrumentation, such as multireflectron devices or complex electric field configurations. In fact, the performance of most simple instruments is likely hindered due to an incomplete understanding of exact ion behaviors. These instruments have the potential to deliver comparable or even superior performance to advanced systems once the optimal ion extraction conditions are realized. This is especially true when it is supported by comprehensive computational approaches, such as the one demonstrated in this study. Moreover, applying this calculation method to conventional instruments is highly promising, provided that the flight-time expression of ions within each ion source geometry can be accurately modeled.

## Conclusion

Using the CSVF model, our calculation results show that a single-stage reflector can achieve a high-order time focusing effect in MALDI R-TOF MS, which was considered impracticable in the past. The model considers ions within a Maxwell-Boltzmann initial velocity distribution to mimic the real condition. According to the results, the delayed extraction technique is much more effective than the reflector on achieving time focusing. Clear evidence was demonstrated by comparing the ion behavior in the continuous and delayed extraction modes. Under continuous extraction mode, the reduction in ions’ flight time spread is insignificant, and no ion focusing point is found at the detector. Under the delayed extraction mode, CSVF calculation can precisely predict the extraction voltage and delay time to achieve first- and second-order time focusing. With first- and second-order focusing conditions, the ions show two focusing points within the instrument. With a regular instrument design, our calculation shows that the best theoretical *R*_*m*_ of *m*/*z* 10,000 ions is above 750,000, or 130,000 if take the detector’s response limit into consideration. The prediction results indicate that some general consensuses and theoretical interpretations are likely inappropriate and need revisions. The results also reveal that a comprehensive calculation method is critically important for further advancement of the instruments to challenge higher performance in a more efficient way.

## Supplementary Material



## Notes

Calculation results of ions with *m*/*z* 1,000 and Spatial and flight-time characteristics of three representative ions in CE and DE modes (PDF)
