# Supplementary material for: Theoretical Study of High-Order Velocity Focusing Achieved with Single-Stage Reflectron Time-of-Flight Mass Spectrometry
Source: J Am Soc Mass Spectrom. 2025 Nov 26;37(1):33–41. doi: 10.1021/jasms.5c00167 (PMC12784402; doi:10.1021/jasms.5c00167)
Supplement: Supplementary file 1 [file js5c00167_si_001.pdf]

Supporting Information

Theoretical Study of High-Order Velocity Focusing Achieved with Single-Stage Reflectron Time-of-flight Mass Spectrometry

*Yi-Hong Cai, Yi-Sheng Wang**

Genomics Research Center, Academia Sinica, Taipei 115, Taiwan.

*Corresponding Author: Yi-Sheng Wang

Email: wer@as.edu.tw

Contents:

A. Calculation results of ions with *m/z* 1,000

Figure S1 The flight-time distribution in the extraction region.

Figure S2 The flight-time distribution in the acceleration region.

Figure S3 The flight-time distribution when reaching the end of the first free-field region.

Figure S4 The flight-time distribution in the reflector.

Figure S5 The flight-time distribution when reaching the exit of the reflector.

Figure S6 The flight-time distribution in the second free-field region.

Figure S7 The flight-time distribution when arriving the detector.

B. Spatial and flight-time characteristics of three representative ions in CE and DE modes

Figure S8 The correlation between position and flight time of three representative ions in DE mode.

Figure S9 Zoom-in views of the results at various positions in Figure S8.

Figure S10 The correlation between position and flight time of three representative ions in CE mode.

Figure S11 Zoom-in views of the results at regions i and ii in Figure S10

A. Calculation results of ions with *m/z* 1,000

The CSVF model was utilized to analyze the time focusing condition of ions of m/z 1,000 in the same instrument as described in the main text. Briefly, the detailed instrument dimensions are: total length = 1,200 mm, extraction region = 8 mm, acceleration region = 10 mm, first field-free region = 882 mmm, reflector = 300 mm, second field-free region = 800 mm. The sample electrode was biased at +25 kV, and the extraction voltage was 200 to 5,000 V. Based on the CSVG model, the optimal extraction voltage for m/z 1,000 is approximately 3,800 V, resulting in a theoretical mass resolving power (R_m) of ~2.87×10^7. The flight-time distributions of the ions in different regions are shown in Figures S1–S7.

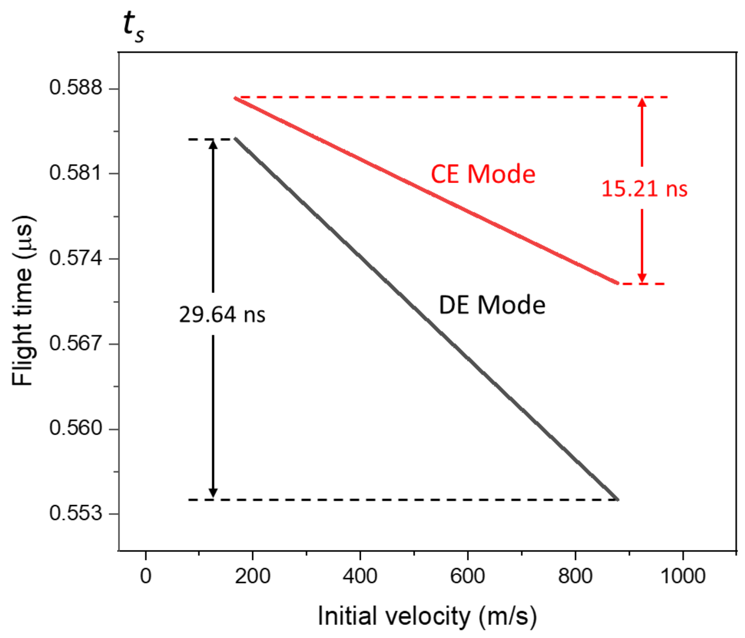

Figure S1 The flight-time distribution of ions under CE and DE modes in the extraction region. Red: CE mode; black: DE mode with a delay time of 540 ns.

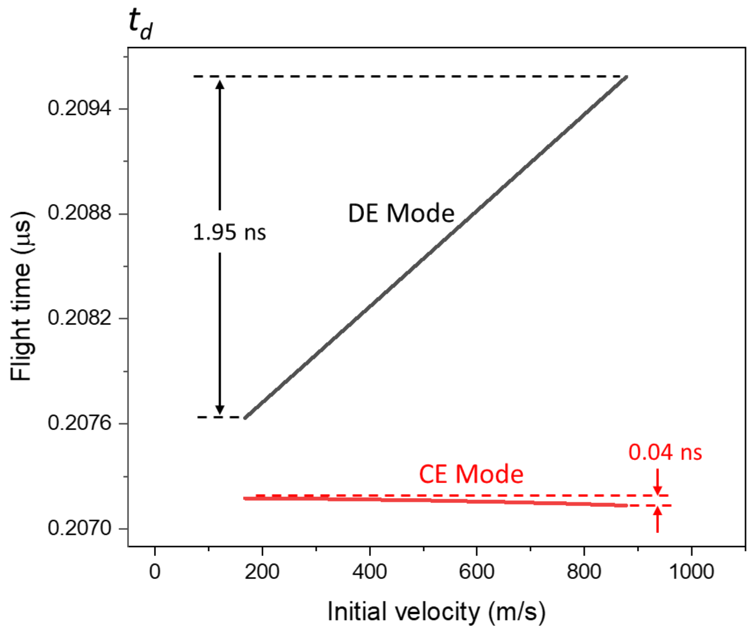

Figure S2 The flight-time distribution of ions under CE and DE modes in the acceleration region. Red: CE mode; black: DE mode (delay time = 540 ns).

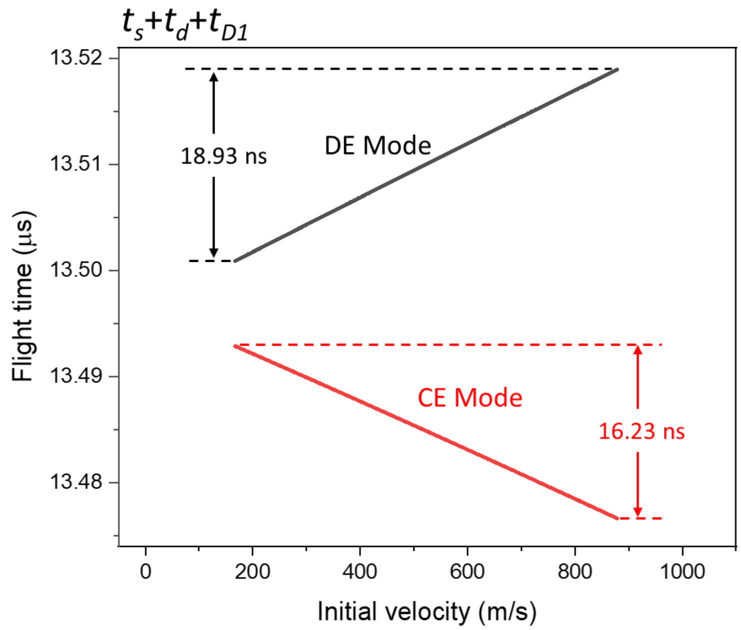

Figure S3 The flight-time distribution of ions under CE and DE modes when reaching the end of first free-field region. Red: CE mode; black: DE mode (delay time = 540 ns).

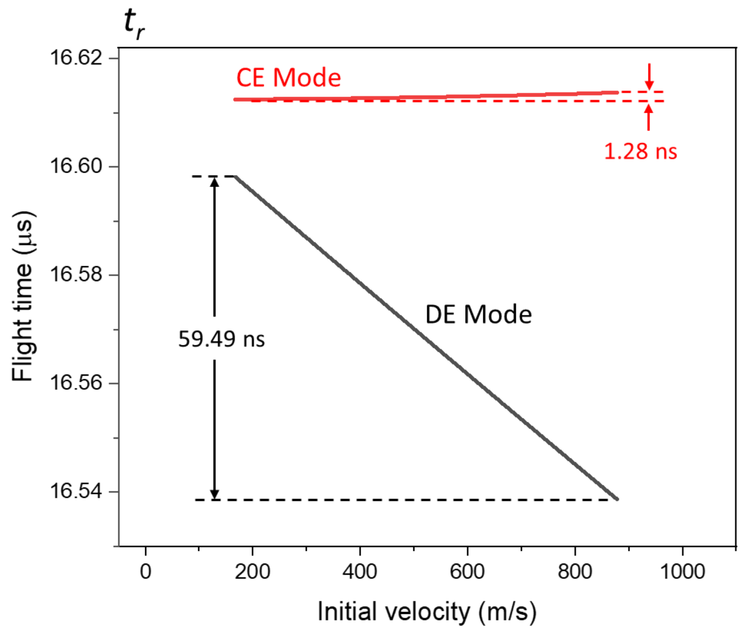

Figure S4 The flight-time distribution of ions under CE and DE modes in the reflector. Red: CE mode; black: DE mode (delay time = 540 ns).

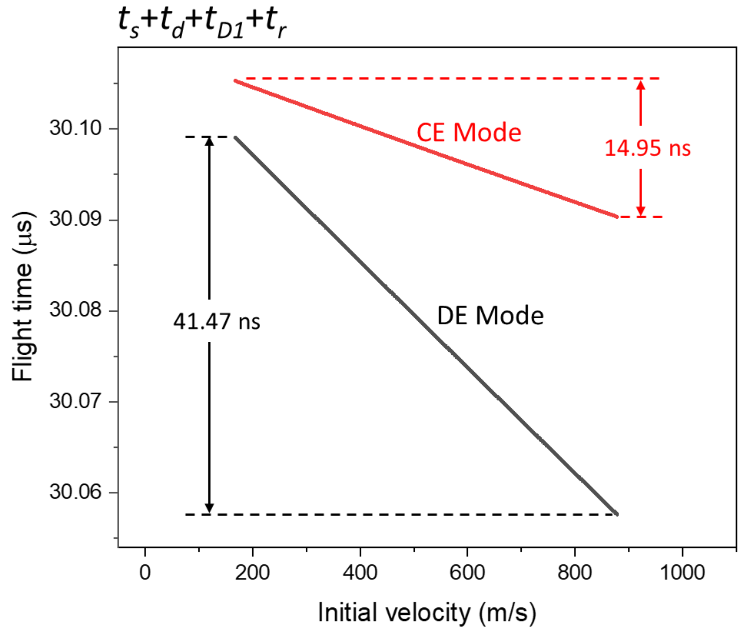

Figure S5 The flight-time distribution of ions under CE and DE modes when reaching the end of the reflector. Red: CE mode; black: DE mode (delay time = 540 ns).

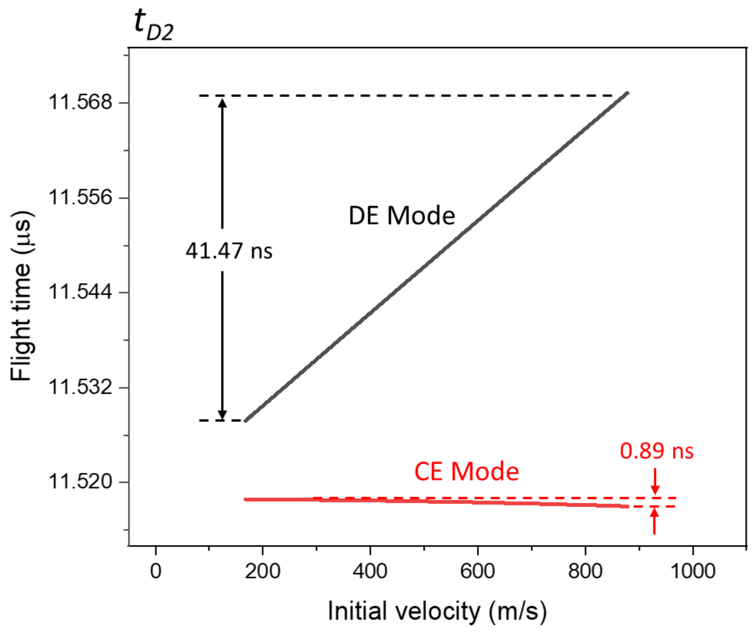

Figure S6 The flight-time distribution of ions under CE and DE modes in the second field-free region. Red: CE mode; black: DE mode (delay time = 540 ns).

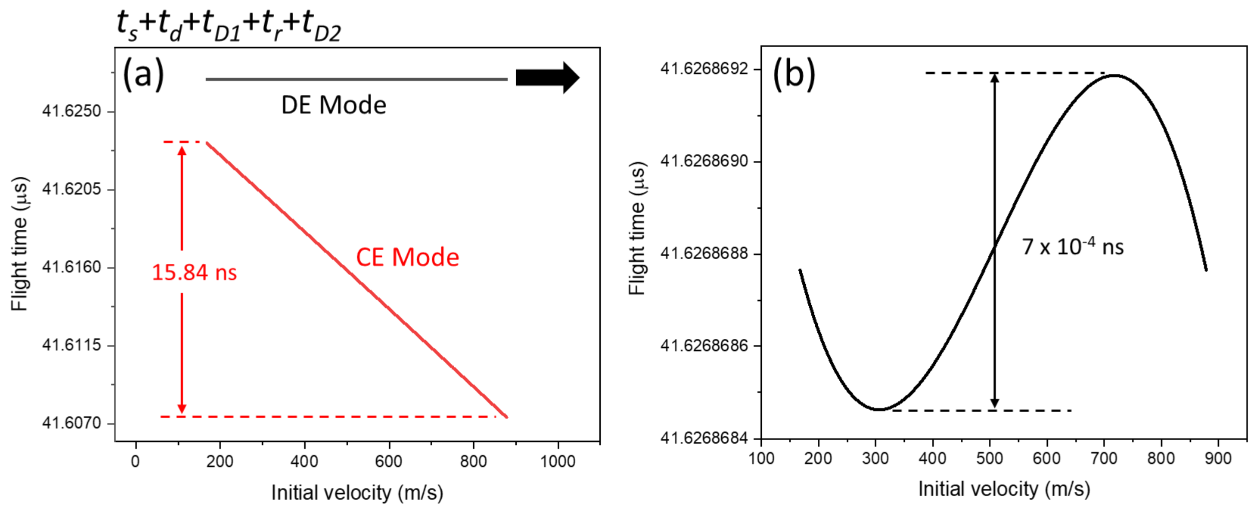

Figure S7 The flight-time distribution of ions under CE and DE modes when reaching the detector. (a) The comparison of CE and DE modes; (b) zoom-in of the data of DE mode. Red: CE mode; black: DE mode (delay time = 540 ns).

B. Spatial and flight-time characteristics of three representative ions in CE and DE modes

The data presented in this section compares the relationship of three representative ions in the instrument. While in the main text Figure 11a and b qualitatively illustrate the relationship and emphasize the differences, the data here show the result quantitatively. The three ions are those with initial velocities of 167 (v_2), 522, and 878 m/s (reference), and with an *m/z* of 10,000. The minimum flight-time spread in the first free-field region is around 2 ns in DE mode, while in the reflector is approximately 26 ns in CE mode.

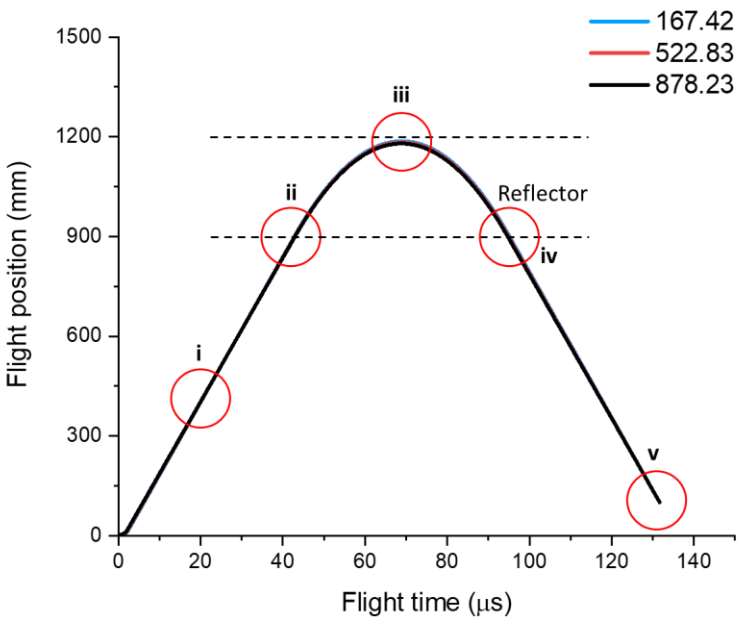

Figure S8 The correlation between position and flight time of three representative ions in DE mode. The three data curves overlapped considerably throughout the instrument. The detailed results in five important regions (i-v) are illustrated in Figures S9.

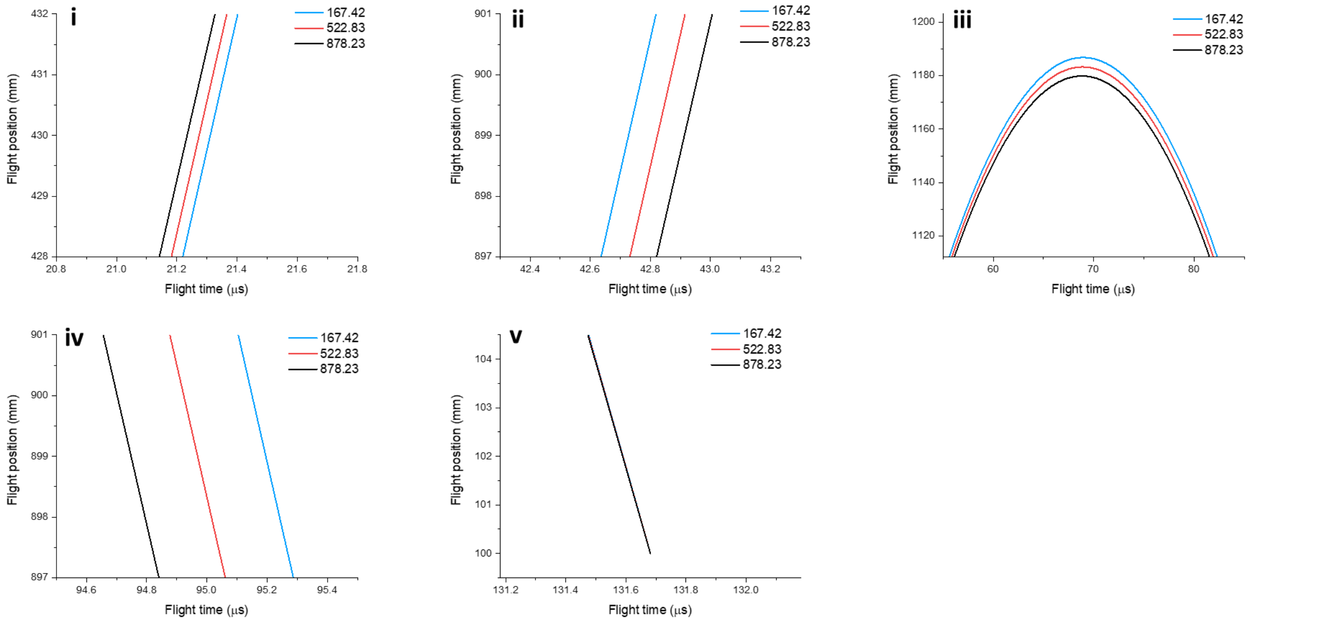

Figure S9 Zoom-in views of the results at various positions in Figure S8. Notably, the flight time sequence of the three ions changed twice, the first one is from regions i to ii, and the second one is from regions iii to iv. The result in the region v shows that the three ions reach the detector almost simultaneously.

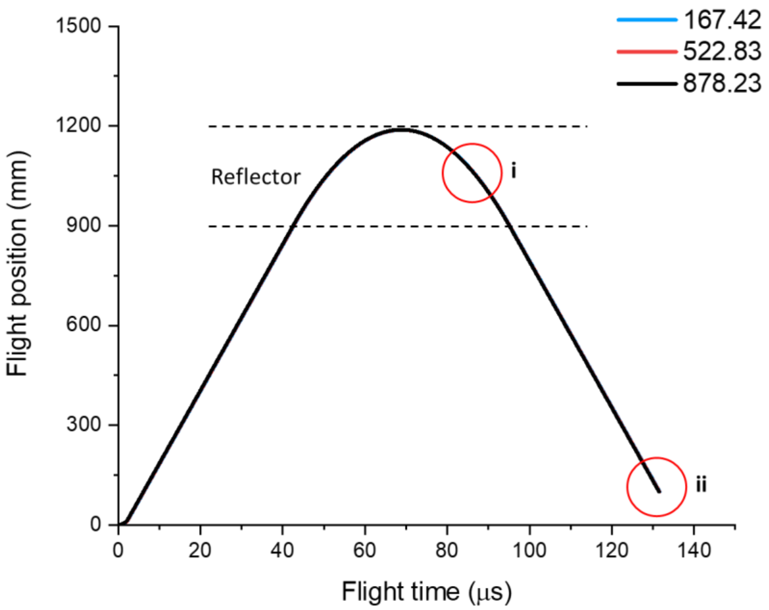

Figure S10 The correlation between position and flight time of three representative ions in CE mode. The three data curves overlapped considerably throughout the instrument. The detailed results in two important regions (i and ii) are illustrated in Figures S11.

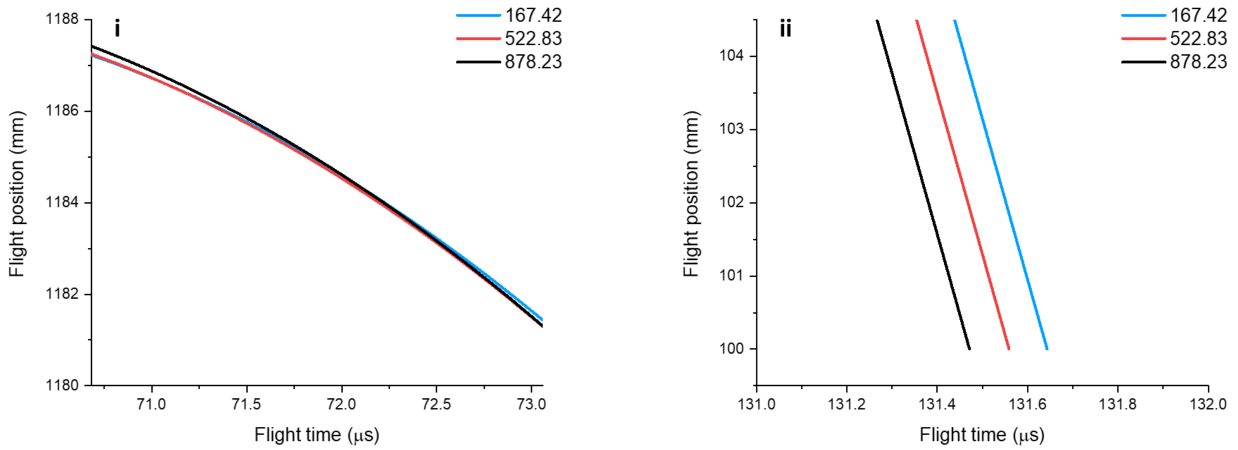

Figure S11 Zoom-in views of the results at regions i and ii in Figure S10. The result in the region ii shows that the three ions reach the detector at different times. The flight time sequence of the three ions did not change throughout the entire instrument. The result in the region ii shows that the three ions reach the detector at different times.
